# Controlled Formation of Nanoribbons and Their Heterostructures via Assembly of Mass-selected Inorganic Ions

**DOI:** 10.1002/adma.202310817

**Published:** 2024-03-05

**Authors:** Xuejiao Zhang, Vesna Srot, Xu Wu, Klaus Kern, Peter A. van Aken, Kelvin Anggara

**Affiliations:** 1 Max-Planck Institute for Solid-State Research, Stuttgart, DE-70569, Germany; 2 Institut de Physique, École Polytechnique Fédérale de Lausanne, Lausanne, CH-1015, Switzerland

**Keywords:** mass-selected ions, ion deposition, self-assembly, nanoribbons, electron microscopy

## Abstract

Control of nanomaterial dimensions with atomic precision through synthetic methods is essential to understanding and engineering of nanomaterials. For single-layer inorganic materials, size and shape controls have been achieved by self-assembly and surface-catalyzed reactions of building blocks deposited at a surface. However, the scope of nanostructures accessible by such approach is restricted by the limited choice of building blocks that can be thermally evaporated onto surfaces, such as atoms or thermostable molecules. We herein bypass this limitation by using mass-selected molecular ions obtained via electrospray ionization as building blocks to synthesize nanostructures that are inaccessible by conventional evaporation methods. As the first example, we show micron-scale production of MoS_2_ and WS_2_ nanoribbons and their heterostructures on graphene, generated by the self-assembly of asymmetrically-shaped building blocks obtained from the electrospray. We expect judicious use of electrospray-generated building blocks would unlock access to previously inaccessible inorganic nanostructures.

## 1 Introduction

One of the hallmarks of nanomaterials is the strong dependence of their properties on their sizes and shapes^1–3^. Owing to quantum confinement effects at the nanoscale, this dependence provides means to tune optical^4–7^ and chemical properties^8–10^ of nanoparticles, as well as means to access unique superconductivity^11^, electrochemical^12^, or light-matter^13,14^ phenomena in monolayer materials^1,2^. As these qualities translate to the strong prospect of nanomaterials to tackle challenges in fields as diverse as energy research^7,15^ and medicine^7,16^, demands to prepare nanomaterials in specific shapes and sizes with atomic precision soar accordingly^3,17–19^. As an example, controlled synthesis of single-layer inorganic nanoribbons comprising a single element^20,21^ or more^22–24^, such as graphene, metal pnictides, chalcogenides, or halides, is a major challenge today that impedes the understanding and application of properties exhibited by these materials^25–28^.

A working strategy to prepare inorganic nanoribbons with selected sizes and shapes employs controlled assembly of smaller building blocks to generate the tailored nanostructures^22–24,29^. Such a ‘bottom up’ approach is widely accomplished by preparing the nanostructures on surfaces^22–24,30^, which takes advantage of confining and steering the assembly process in two dimensions via surface structures and surface-catalyzed reactions. Despite its widespread use, this strategy, however, relies on the transfer of building blocks from a source onto the surface by thermal evaporation^31–34^, which restricts the building blocks to the ones that are sufficiently volatile and stable at high temperatures, such as atoms or simple molecules. The constraint on the choice of usable building blocks limits the range of nanostructures accessible by such approach.

A way to sidestep this limitation involves the use of electrospray ionization^35^ to transfer non-volatile, thermolabile building blocks as intact ions into the gas phase, before selecting and landing these ions intact on surface for their subsequent assembly into nanostructures^36–39^. The use of such strategy for selected inorganic ions to prepare inorganic nanostructures on surfaces has so far been shown to yield inorganic clusters^40–44^, nanoparticles^45^, and multilayers mimicking electrode-electrolyte interfaces^46–48^. Yet, due to the vast selection of usable building blocks, the soft landing technique remains to be an attractive tool to access the uncharted chemical space of inorganic nanostructures. For example, building blocks terminated with specific structures could yield nanostructures with tailored atomic termination that gives unique contact resistance or Schottky barrier.

Here, we extend the scope of ion soft landing technique in the field of inorganic nanostructure synthesis. We show that, by allowing precise selection of complex molecular building blocks and control of their deposited quantities on surface, the soft landing technique unlocks opportunities to prepare inorganic nanostructures that are presently inaccessible by conventional methods. As the first example, we show the formation of MoS_2_ nanoribbons from mass-selected MoS ions (HS(MoS_3_)_N_^1-^, N = 4 to 8) soft landed on a freestanding, single-layer graphene at ambient temperature *in vacuo* by the Electrospray Ion Beam Deposition (ESIBD)^36,39^ technique (see Methods). Atomic level structural characterization of the nanoribbons by Scanning Transmission Electron Microscopy (STEM) corroborated by Density Functional Theory (DFT) calculations attributes the formation of MoS nanoribbons to the molecular asymmetry of the unique MoS building blocks deposited on the surface. We further show the perspective of the technique by using two different building blocks, MoS and WS ions, to generate two-component core-shell heterostructures and alloyed nanostructures. The precise control of deposited building blocks and their quantities enabled by the method permits dimensional and stoichiometry control of the resulting nanoribbons at a surface, thereby providing new systems to explore nanoribbon electronics^28^ and photonics^14^, as well as a new synthesis strategy to the burgeoning field of two dimensional material synthesis.

## 2 Results and discussion

### 2.1 Nanoribbons formation by assembly of molecular building blocks

**Figure 1:**
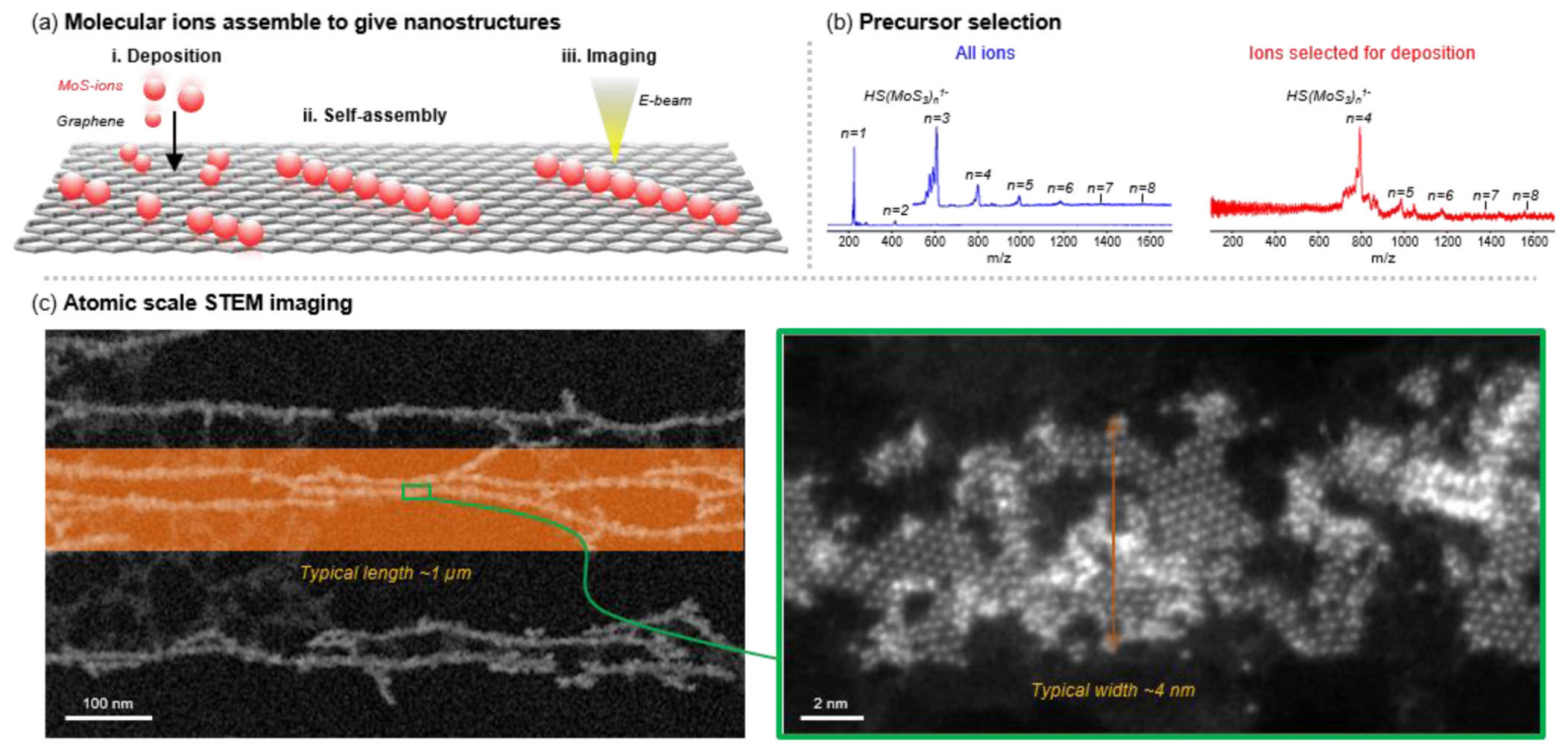
MoS ions deposited on graphene assemble to give single-layer MoS_2_ nanoribbons. (a) Mass-selected MoS ions were soft-landed with 3 eV landing energy at a single-layer graphene by Electrospray Ion Beam Deposition (ESIBD) and assembled into MoS_2_ nanoribbons as observed by Scanning Transmission Electron Microscopy (STEM) (see Methods). (b) Time-of-flight mass spectrum of the MoS ions obtained from electrospray (blue) and mass-selected MoS ions used for surface deposition (red), showing a series of [HS(MoS_3_)_N_]^1–^ ions (N = 4 to 8). (c) The MoS_2_ nanoribbons were imaged at the atomic level by High-Angle Annular Dark-Field (HAADF) STEM imaging, showing a typical length of ~1 μm and width of ~4 nm.

Mass-selected MoS ions (HS(MoS_3_)_N_^1-^, N = 4 to 8) deposited on graphene were observed to form single-layered MoS_2_ nanoribbons by using STEM in High-Angle Annular Dark-Field (HAADF) mode (Figure 1), whose contrast is proportional to the thickness and the atomic mass of the object. We observed the entire series of HS(MoS_3_)_N_^1-^ from N = 1 to 8 only when a relatively high concentration of (NH_4_)_2_MoS_4_ solution (~10 mM) was electrosprayed, by taking advantage of the notion that high concentration promotes cluster formation^49,50^. Out of the observed MoS ion series, only HS(MoS_3_)^1-^ and HS(MoS_3_)3^1-^ have been previously observed, where HS(MoS_3_)^1-^ is a typical ion observed from solutions of MoS_4_^2-^ salts^51^, while HS(MoS_3_)_3_^1-^ has been observed from gas phase dissociation of HMo_3_S_13_^-^ ions^52^. We chose to study heavy MoS ions (HS(MoS_3_)_N_^1-^, N ≥ 4) due to their higher adsorption energies on graphene, which allow them to adhere to graphene stronger and to be retained on graphene long enough to form the nanoribbons (Figure S1 and S2).

The nanoribbons were measured to be few microns in length and few nm in width with a significant amount of edges (a ~4-fold increase in edge length when compared to a solid nanoribbon with same dimensions, see Methods for analysis) that are ideal for catalysis^53^ and nanoscale electronics^54^. We find that the high number of step edges in these nanoribbons is significantly different from MoS_2_ nanoribbons fabricated by electron beam irradiation^55^ or by droplet evaporation^23^, which opens new opportunities to explore the chemistry and electronic properties of these step edges.

**Figure 2:**
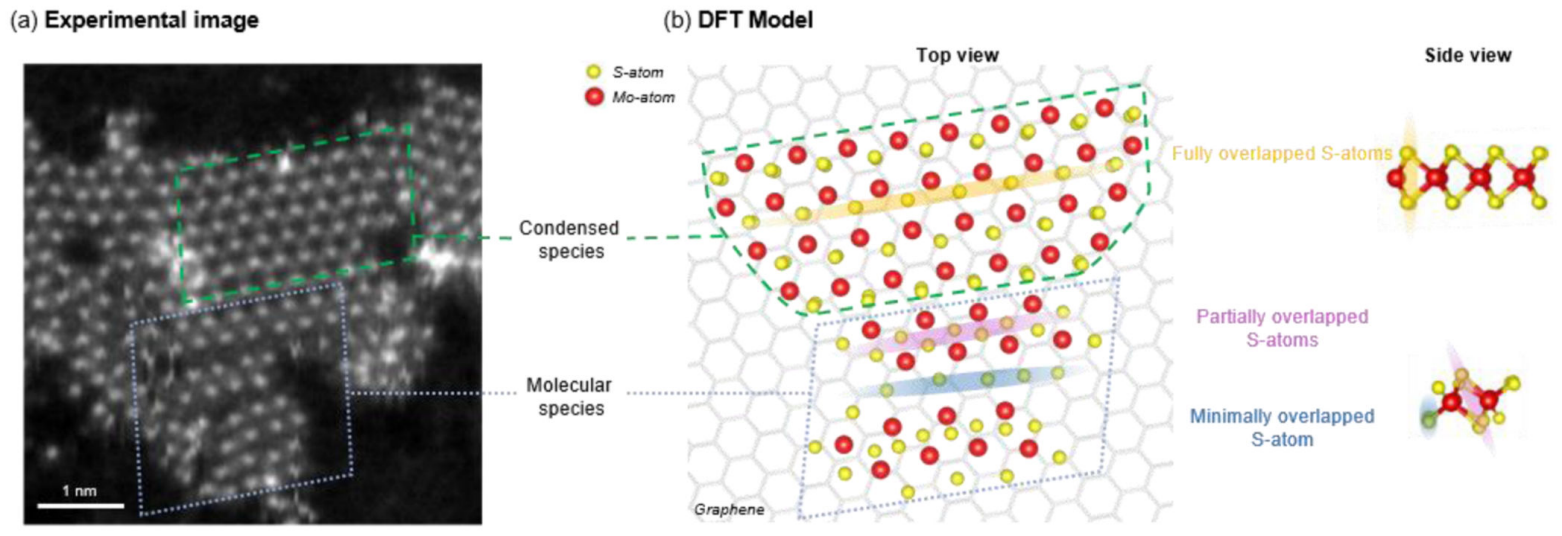
Detailed structures of the MoS_2_ nanoribbons revealed by STEM-HAADF imaging and DFT calculations. (a) Atomic-resolved STEM-HAADF image reveals ‘condensed’ 1H-MoS_2_ domains (green box) surrounded by coalesced ‘molecular’ MoS_2_ (blue box). The image shows one typical contrast for Mo atoms and three different contrasts for S atoms (see Figure S5 for details), indicating three distinct geometries of S atoms in the nanoribbons. (b) Computed structures obtained from the Density Functional Theory (DFT) calculations interprets the STEM-HAADF images to unveil the atomic structures of ‘condensed’ and ‘molecular’ MoS_2_ observed in the nanoribbons.

We confirmed the single-layer nature of the nanoribbons by observing similar HAADF contrast between the Mo atoms in the nanoribbons and single Mo adatoms on graphene (Figure S3), evidencing that the nanoribbons were one atom thick. The presence of Mo and S in the nanoribbons was confirmed by the characteristic Mo peaks observed in energy-dispersive X-ray (EDX) spectroscopy (Mo-Kα and Mo-Lα) and S-L_2,3_ edges observed in Electron Energy-Loss Spectroscopy (EELS) (Figure S4a). Combining these results with the analysis of our STEM-HAADF images (Figure 2), we confirmed the presence of hexagonal MoS_2_ (1H-MoS_2_) in the nanoribbons, as evidenced by the symmetry and the measured Mo-Mo distance of 3.15 ± 0.05 Å matching those for 1H-MoS_2_ (3.15 Å)^56^. From these results, we conclude that the MoS_2_ nanoribbons are formed by a quasi-1D assembly of deposited MoS molecules on graphene, which we note to deviate significantly from the typical triangular growth pattern of MoS_2_ island obtained from vapor deposition techniques^57^. Our findings hence underscore the importance of building blocks in dictating the growth mechanism and the final nanostructures on surface.

To uncover the formation mechanism of the MoS_2_ nanoribbons, we subsequently examined the effect of surface structures on the assembly of MoS ions into the nanoribbons. Remarkably, the nanoribbon formation was found to be strongly affected by the surface, since we only observed the nanoribbons on clean single-layer graphene, but not on contaminated single-layer graphene, bilayer graphene, or amorphous carbon. Assembly of MoS ions on single-layer and bilayer graphene contaminated with hydrocarbons^58^ was observed to yield shorter nanoribbons with greater number of branches, whereas, on amorphous carbon surfaces, randomly shaped MoS islands were observed (Figure S5). These results indicate that rough surfaces inhibit the nanoribbon formation and instead promote the formation of branches, while flat surfaces, such as clean single-layer graphene, promote the nanoribbon formation. We verified that the single-layer graphene used in our experiments was flat by confirming the absence of graphene ripples^59^ in Scanning Electron Microscopy (SEM) images of our graphene samples (Figure S6). We further confirm that the absence of graphene grain boundaries in our samples (Figure S7), ruling out these line defects as the origin of the nanoribbon orientation (Figure S8).

Further clues to the nanoribbon formation mechanism are revealed by STEM-HAADF imaging, which unveils the presence of adsorbed ‘molecular’ MoS_2_ that would play a key role in the nanoribbon formation. The ‘molecular’ MoS_2_ was identified from nanoribbon structures resolved at the atomic level by STEM-HAADF imaging and corroborated by DFT calculations (Figure 2). While the Mo atoms were observed with a single HAADF contrast evidencing the monolayer nature of the nanoribbon, the S atoms were observed with three different HAADF contrasts (Figure S9). These three contrasts were interpreted by DFT calculations to be three different geometries of S atoms when observed from the top (Figure 2b): the bright contrast is due to two fully overlapped S atoms, the dim contrast is due to two partly overlapped S atoms, and the dark contrast is due to minimally overlapped S atoms. Consequently, the interpreted nanoribbon structures allow the ‘molecular’ MoS_2_ to be distinguished from the ‘condensed’ domain of 1H-MoS_2_ (Figure 2). The ‘molecular’ MoS_2_ features an S atom coordinated by two Mo atoms along its edge, and at its interior, an S atom coordinated by three Mo atoms (Figure S10), similar to the MoS_3_ intermediate in ref 60. These distinct S atom coordinations may provide a new strategy to tailor contact resistance and Schottky barrier in metal-semiconductor junctions. As we detail below, the ‘molecular’ MoS_2_ is understood to be an important precursor to the ‘condensed’ MoS_2_, and thus the MoS_2_ nanoribbon.

### 2.2 Formation mechanism of nanoribbons

To account for the observation of ‘molecular’ MoS_2,_ and to clarify its origin and role in the nanoribbon formation, we performed *ab initio* Molecular Dynamics (MD) calculations (see Methods) to model the deposition and assembly of MoS molecules at a flat graphene surface. We first examined the deposition event by computing the most stable structure of the MoS ions in the gas phase and its subsequent landing dynamics on graphene. We chose the Mo_8_ species as a model system to study the chemical reactivities of the different S-terminations present in Mo_8_S_16_ and HMo_8_S_25_ species. The most stable gas-phase structure of HMo_8_S25^1-^ ion was computed to strongly resemble the corresponding ‘molecular’ Mo_8_S_16_ on graphene (Figure S10) with the only difference being: in ‘molecular’ MoS_2_, two edge Mo atoms are bridged by *one* S atom, while in MoS ions, the two edge Mo atoms are bridged by *two* S atoms. This resemblance indicates that the ‘molecular’ MoS_2_ could be derived from the adsorbed MoS ions, as corroborated by our calculations revealing exothermic reaction pathways for ‘molecular’ MoS_2_ to be produced from the reaction between adsorbed MoS ions and chemisorbed S atoms, observed on graphene (Figure S3). Notably, while HMo8 S25 ^1-^ ion is understood to possess a charge of -1*e* in the gas phase, Bader analysis of the adsorbed HMo_8_S_25_ on graphene shows that the molecule has a charge of -0.05*e*. This result indicates that the adsorbed MoS ions are better understood as a neutral molecule on graphene as a result of the gas-phase MoS ions losing an electron upon adsorption to graphene. We expect that such electron transfer^61^ is possible, because the energy of the filled molecular orbitals of the gas-phase MoS ions is higher than the Fermi level of the graphene.

The computed exothermic pathways linking HMo_8_S_25_ to Mo_8_S_16_ (Figure S11) show chemisorbed S atoms (E_ads_ = 1.8 eV) attaching to the S_2_ edge of HMo_8_S_25_ to give S_3_ or S_4_ edges that dissociate to leave an S_1_ edge on the MoS molecule and weakly physisorbed S_2_ (E_ads_ = 0.3 eV) or S_3_ (E_ads_ = 0.4 eV), which we expect would desorb from graphene due to their low adsorption energies. Further conversion of all S_2_ edges in HMo_8_S_25_ to S_1_ edges thereby converts HMo_8_S_25_ to Mo_8_S_16_, which belongs to the ‘molecular’ MoS_2_ family and is expected to diffuse and assemble into nanoribbons. We note, however, that while energy considerations are important in such reactions, activation barriers and entropy may also play a role. Further support for the formation of ‘molecular’ MoS_2_ via reactions on the surface is given by our MD calculations showing that the structure of HMo_8_S_25_ is preserved upon its landing on graphene (Figure S12), thereby ruling out molecule-graphene collision as a mechanism for ‘molecular’ MoS_2_ formation.

To understand how ‘molecular’ MoS_2_ assemble into nanoribbons, we model the assembly of ‘molecular’ MoS_2_ on graphene at room temperature by simulating collisions between two ‘molecular’ MoS_2_ at varied angles and impact parameters (i.e. miss distance between two approaching objects) (see Methods). Based on our MD calculations examining 100 different collision geometries, collisions between two ‘molecular’ MoS_2_ lead to either formation of a ‘condensed’ MoS_2_ island, or a network of coalesced ‘molecular’ MoS_2_ (Figure S13), consistent with our experimental observations (Figure 2). In the formation of ‘condensed’ MoS_2_, two ‘molecular’ MoS_2_ collide to form multiple new Mo-S bonds to yield a tetragonal MoS_2_ island (1T-MoS_2_), which, at longer timescales, is expected to spontaneously convert to a thermodynamically more stable hexagonal MoS_2_ island (1H-MoS_2_) observed in experiments. In the formation of coalesced ‘molecular’ MoS_2_ network, two ‘molecular’ MoS_2_ collide to form a limited number of Mo-S bonds that unite the two molecules, yielding a network of coalesced ‘molecular’ MoS_2_ observed in experiments. Our MD calculations, by giving ‘condensed’ and ‘molecular’ MoS_2_ outcomes are consistent with the experimental observations, providing a basis to further understand the formation mechanism of MoS_2_ nanoribbons.

The formation of MoS_2_ nanoribbons is understood to be due to preferential assembly of ‘molecular’ MoS_2_. By analyzing how ‘molecular’ MoS_2_ interacts with one another from our STEM-HAADF images (N = 636 pairs, see Methods), a pair of ‘molecular’ MoS_2_ was found to prefer a head-to-head contact (50%) than head-to-side contact (33%) or side-to-side contact (16%). These results are corroborated by our MD calculations, which show that head-to-head collisions almost always result in a reactive outcome (95%, 19 out of 20 trajectories), while head-to-side and side-to-side collisions result in less reactive outcomes (85%, 46 out of 54 trajectories; and 83%, 20 out of 24 trajectories, respectively). Our findings in both experiments and calculations hence suggest an increased reactivity of ‘molecular’ MoS_2_ at its terminal edges than its side edges. The anisotropic reactivity of ‘molecular’ MoS_2_ is understood to drive the anisotropic growth of MoS_2_ island into MoS_2_ nanoribbons observed in experiments.

### 2.3 Fine tuning of nanoribbon structures

**Figure 3:**
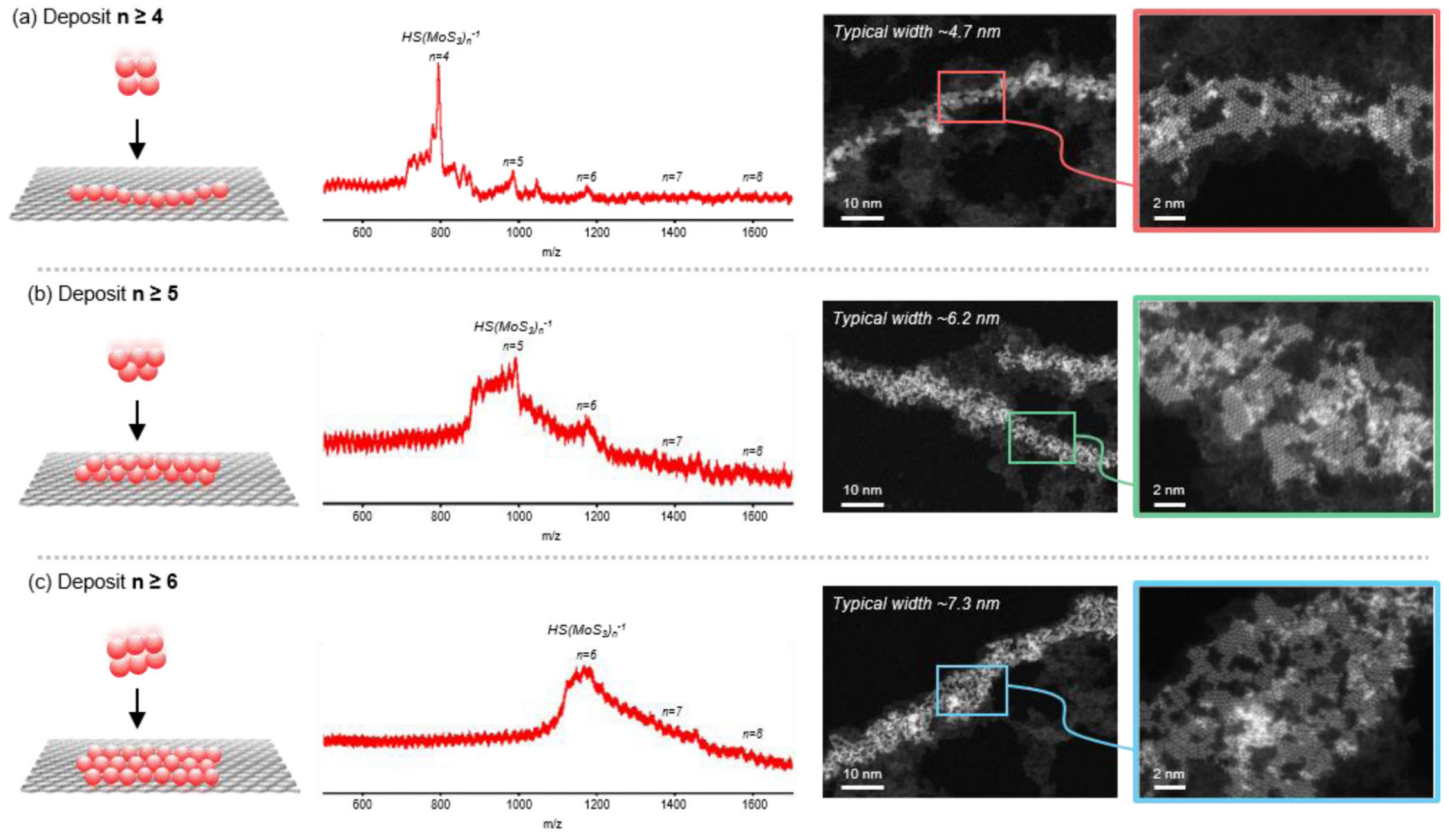
Varying deposited MoS ions tunes the width of formed MoS_2_ nanoribbons. Varying deposited [HS(MoS_3_)_N_]^1–^ ions on graphene from N ≥ 4 in (a), N ≥ 5 in (b) to N ≥ 6 in (c) increases the average width of the resulting nanoribbons from 4.7 nm to 7.3 nm.

The formation of MoS_2_ nanoribbons from the anisotropic assembly of asymmetric MoS building blocks informs a means to tune the final nanoribbon structures by the MoS species deposited on surface. We demonstrate this capability by varying the selected MoS ions (HS(MoS_3_)_N_^1-^) deposited on graphene, starting from N ≥ 4 to N ≥ 6 (Figure 3). In all cases, we observed monolayer MoS_2_ nanoribbons with similar porosity, albeit with different average widths. The porosity of the nanoribbons, determined for all three samples to be ~2 nm edge length per 1 nm^2^ of the nanoribbon area, were found to be approximately 4-fold larger than that for a solid nanoribbon (see Methods for detailed analysis). The widths of the nanoribbons were found to vary from 4.7 ± 1.7 nm for the case of N ≥ 4; 6.2 ± 2.2 nm for N ≥ 5; to 7.3 ± 2.8 nm for N ≥ 6 (see details in Figure S14). In addition, we found that the quantity and the length of nanoribbons on the surface could be increased without varying their widths by increasing the amount of deposited MoS ions, as observed in samples with low and high coverage of MoS ions (Figure S15). These findings thereby highlight the importance of controlling the building block species and their deposited quantities to tune the final state of the nanostructures on surface.

### 2.4 Two-component nanoribbon heterostructures

**Figure 4:**
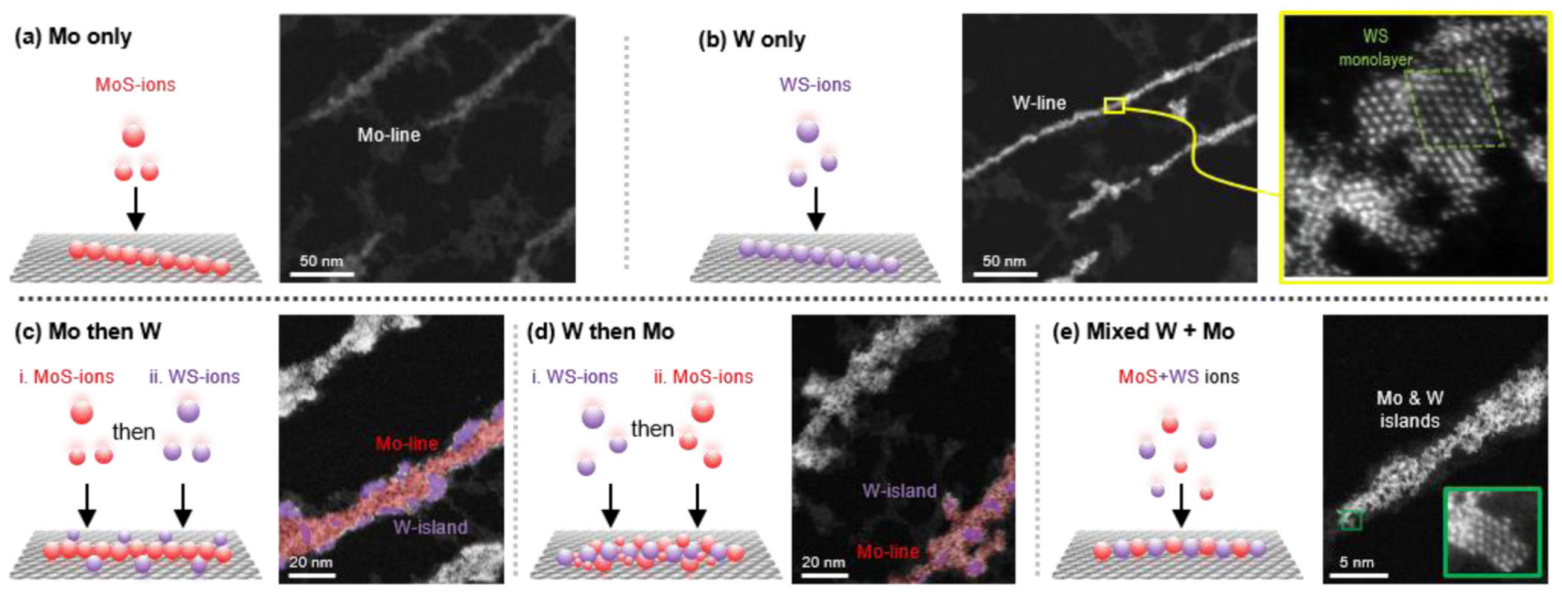
Deposition of MoS and WS ions on graphene yields various MoS- and WS-based core-shell and alloyed heterostructures. Both MoS ions [HS(MoS_3_)_N_]^1–^ (N ≥ 6) and WS ions [HS(WS_3_)_N_]^1–^ (N ≥ 4) are respectively found to assemble on graphene into MoS_2_ nanoribbons (a) and WS_2_ nanoribbons (b). Sequential deposition of MoS ions followed by WS ions is found to generate core-shell heterostructures with an MoS-core and a WS-shell (c), whereas the deposition of WS ions followed by MoS ions results in the same core-shell structures with a WS-core and an MoS-shell (d). Concurrent deposition of MoS and WS ions results in alloyed nanostructures containing Mo, W, and S (e). Detailed imaging in the inset shows the substitution of Mo atoms by W atoms, and vice versa, across the lattice.

To illustrate the perspective of the technique, we show that soft landing of mass-selected ions enables morphology control of nanoribbons consisting of two transition metals (Figure 4). We exemplify this for two-component (Mo and W) nanoribbons prepared by co-depositing MoS ions and WS ions on graphene. In the co-deposition experiment, we chose to deposit WS ions with a chemical formula similar to that for MoS ions, namely HS(WS_3_)_N_^1-^ (Figure S16). As a result, the WS ions (N ≥ 4) assembled into WS_2_ nanoribbons (Figure 4b), similar to the MoS ions (Figure 4a). We confirmed the presence of W and S on the WS nanoribbons using EDX which revealed the characteristic W peaks (W-Lα) and S peaks (S-Kα) (Figure S4b). The nanoribbons obtained from assembled WS ions suggest an assembly mechanism similar to that for MoS ions, which we exploited herein in our co-deposition experiments to keep the assembly mechanism as a constant.

The MoS and WS co-deposition experiments were observed to yield nanoribbon heterostructures with different morphology, depending on whether the building blocks were deposited sequentially (Figure 4c,d) or concurrently (Figure 4e). Sequential deposition of one type of ions followed by another type of ions yielded nanoribbons with a core-shell structure, be that MoS nanoribbons with WS shells (Figure 4c) or WS nanoribbons with MoS shells (Figure 4d), as confirmed by EDX (Figure S4c,d). The core-shell structure suggests that the nanoribbon ‘core’ formed by the first building blocks provides favorable nucleation sites for the second building blocks to form the ‘shell’ of the nanostructure. In contrast, concurrent deposition of the two types of ions (Figure S17) yielded alloyed nanoribbons with both Mo and W atoms incorporated into the nanoribbons (Figure S4e). The MoWS-alloy structures were observed as W atoms substituting those sites, where Mo atoms were expected and vice versa (Figure 4e inset), suggesting that the MoS and WS building blocks assemble together to yield the observed alloyed nanoribbons. The co-deposition of MoS and WS building blocks, either sequential or concurrent, demonstrates a new means to prepare lateral heterostructures (or heterojunctions) of monolayer materials^62–65^ or high entropy monolayer materials^66^ using non-volatile, molecular building blocks.

## 3 Summary and outlook

The assembly of non-volatile molecular building blocks enabled by soft landing of inorganic ions on single-layer graphene using the ESIBD technique is demonstrated to yield inorganic nanoribbons with unique and tunable composition, stoichiometry, and morphology as confirmed by atomic-resolved STEM-HAADF imaging. Unlike conventional vapor deposition techniques, our approach of soft landing mass-selected ions on surfaces provides control over the species of the building blocks and their quantities on surface. As an example, the present work shows the use of elongated building blocks to prepare a diverse range of 2D nanostructures, such as nanoribbons, as well as core-shell and alloyed nanoribbons. The use of ionic building blocks from the electrospray ionization allows the selection of building blocks down to specific isotopes or oxidation states, the use of *in vacuo* generated species^67^, the reduction of materials needed for deposition to few micromoles (μmol), and the use of less-hazardous and air-stable transition metal salts as transition metal sources. In addition, the use of electrospray opens the possibility to use of custom building blocks prepared by wet inorganic or organometallic synthesis^68,69^, as well as to integrate the field of 2D material synthesis to the high throughput electrospray-based technologies in combinatorial^69,70^ or bioanalytical chemistry^71^. By bringing in techniques from various fields of chemistry, we anticipate that our method would be capable of providing synthetic access to a greater range of 2D materials that remain intractable at present by conventional thermal-based methods, and thus open new opportunities in structure-properties studies of 2D materials.

## Methods

### Surface preparation

Clean single-layer graphene was prepared closely following previously established procedures^72^ using the PMMA-coated CVD-grown graphene on a Cu-foil (Graphenea S.A.) (PMMA = polymethyl methacrylate, CVD = chemical vapor deposition). Briefly, the Cu-foil was removed by placing the PMMA-graphene-Cu film in an etchant solution made of 8 g of ammonium persulfate in 100 mL of Milli-Q water. Following the Cu-removal, we removed any remaining etchant residue on the PMMA-graphene stack by rinsing in a Milli-Q water. The stack was subsequently transferred onto a holey SiN_x_ support grid (Ted Pella, Inc., Catalog#: 21581-10) with the graphene-side being in contact with the grid. Prior to the transfer, the SiN grids featuring 1 μm diameter holes across the membrane were sputter coated with ~10 nm of Pt that would catalyze the PMMA removal. To prepare the clean single-layer graphene for the deposition experiment, the PMMA was removed from the PMMA-graphene stack on the SiN grid by annealing the grid in ambient atmosphere at 300 °C for 30-60 min, whereupon the Pt sputtered on the grid catalyzed the oxidation of the PMMA. Clean single-layer graphene was consistently obtained by transferring the grid into vacuum chambers, while the grid was hot (within 2-3 min after annealing). On the other hand, to obtain the single-layer graphene contaminated with hydrocarbon impurities^58^, the annealed graphene-on-SiN-grid was transferred into the vacuum chambers after it cooled to room temperature. The contaminated bilayer graphene was obtained as the bilayer graphene impurities present in the starting material of our single-layer graphene samples. The holey carbon surface (Quantifoil TEM substrate) was obtained commercially and used without further treatment (Ted Pella, Inc., Catalog#: 656-200-CU).

### Ion deposition

Ammonium tetrathiomolybdate, (NH_4_)_2_MoS_4_, (99.97%, Sigma Aldrich, Catalog#: 323446, CAS: 15060-55-6) and ammonium tetrathiotungstate, (NH_4_)_2_WS_4_, (≥99.9%, Sigma Aldrich, Catalog#: 336734, CAS: 13862-78-7) were used without further purification. For the electrospray ionization, we used (NH_4_)_2_MoS_4_ or (NH_4_)_2_WS_4_ solutions with an elevated concentration (10 mM in 1:1 water:isopropyl-alcohol) to promote the condensation of the Mo or W species in the generated electrospray microdroplets, as suggested by Refs 49 and 50. Using the ESIBD technique, described in detail elsewhere^73^, we characterized the negative ions obtained from the electrospray using a home-built time-of-flight mass spectrometer, mass-selected the desired ions using the quadrupoles, and aimed the mass-selected ions onto the surface (e.g. graphene on a TEM grid) held at room temperatures in a high vacuum (HV) chambers of 10^-7^ mbar. We ensured the intact landing of the deposited MoS or WS ions by applying a voltage on the surface which would decelerate the incident ions to a low kinetic energy of ~3 eV, considered to be well in the range of ‘soft landing’^36,74^. Following the deposition, the sample was transferred to an HV load lock chamber, which was subsequently vented with dry nitrogen gas before the sample was taken out into ambient atmosphere and transferred into the STEM instrument for the imaging experiment *in vacuo*. Ion currents and deposition times in our experiments varied between 10-50 pA and 20-240 minutes, according to the desired coverage and the ion species (Table S1).

### HAADF-STEM imaging

STEM imaging was performed with an aberration-corrected JEOL ARM200F STEM instrument, equipped with a cold-field emission gun, a spherical aberration corrector (DCOR, CEOS GmbH), an energy dispersive X-ray detector (JEOL), a GIF Quantum ERS electron energy-loss spectrometers (Gatan), and a GIF CCD camera (Gatan). The HAADF-STEM images were acquired by JEOL ADF detector with a convergent semi-angle of 33.5 mrad and collection semi-angles of 56–234 mrad, with 16 μs/pixel dwell time. The EDX spectra were acquired at 5200 cps with 300 s live time for each spectrum. Several scans were performed on the same area to obtain a cumulative spectrum with a better signal-to-noise ratio. For EELS experiments, a pixel dwell time of 0.02 s and an energy dispersion of 0.25 eV/channel (resulting in an energy resolution of 1 eV) were used, and the spectra were acquired with a convergent semi-angle of 33.5 mrad and collection semi-angle of 85 mrad for a 5 mm aperture. All measurements were performed at an acceleration voltage of 60kV of the electron beam.

### SEM imaging

The SEM images were acquired with an FEI SCIOS SEM instrument at 50 nA, 30 kV, and dwell time of 16 μs per pixel. The sample was tilted more than 70° to check for any graphene ripples on our graphene samples.

### *Ab initio* calculations

DFT calculations were performed to model the experimental observations with parameters closely following values well known to reproduce the experimental results^74^. All structures were visualized using the VESTA software^75^. To model observations on graphene, the plane-wave based Vienna Ab-initio Simulation Package code (VASP, version 5.4.4)^76,77^ was used, employing the projection-augmented wave function (PAW) method^78,79^ with a cut-off energy of 400 eV, the Perdew-Burke-Ernzerhof (PBE) functional^80^, and the Grimme’s DFT-D3 approach^81^. All calculations were performed in a supercell with 30 Å vacuum space by sampling only the gamma point of the k mesh. The relaxation calculations were performed until the forces were below 0.01 eV/Å for all atoms. The adsorption energies (E_ads_) were defined as the energy released when a molecule was adsorbed at a surface (positive value means the adsorption process is exothermic). The charge of the molecule on graphene was analyzed by the Bader’s atom-in-molecule formalism^82^ using the Henkelmann algorithm^83^. The MD calculations were run as a microcanonical ensemble preserving the number of atoms (N), the volume (V), and the energy (E). For the landing dynamics calculations, HMo_8_S_25_ molecule was placed ~15 Å above the graphene and had the velocities of all atoms initialized by random velocities sampled from the Boltzmann distribution at 298 K. In addition to these velocities, every atom in the MoS molecule was added with a velocity corresponding to 3 eV of molecular translational energy toward the graphene. For the collision dynamics calculations, two ‘molecular’ Mo_8_S_16_, one as the target and another as the projectile, were placed on graphene with various angles and impact parameters, and had the velocities of all atoms initialized by random thermal velocities at 298 K. To approximate the thermal reaction between the two molecules, we added ~90 meV of translational energy to every atom in the projectile MoS_2_ molecule towards the target MoS_2_ molecule. To model the gas-phase MoS ions, the ORCA code (version 5.0.3)^84^ was used, employing PBE functional^80^, DFT-D3 correction^81^, and the ma-def2-SVP basis sets^85,86^ with auxiliary basis sets chosen automatically^87^.

### Image analysis

Prior to analysis, all images were calibrated using the lattice parameters of single-layer graphene observed by STEM imaging. Distances were measured using the WSXM software^88^, and all errors reported in the paper are the standard deviation. For the analysis of ‘molecular’ MoS_2_ on the surface, we categorized interacting pairs of ‘molecular’ MoS_2_ as either head-to-head (HH), head-to-side (HS), or side-to-side (SS) interactions. First we identified interacting pairs of ‘molecular’ MoS_2_ on surface based on their Mo-Mo distances. For a pair of ‘molecular’ MoS_2_ i.e. molecules A and B, we considered that the molecules were interacting if any Mo atom in molecule A (Mo_A_) was found below 4.4 Å away from any Mo atom in molecule B (Mo_B_). The 4.4 Å threshold was determined by measuring the typical nearest distance between Mo_A_ and Mo_B_ in a pair of ‘molecular’ MoS_2_ adjacent to each other. Following this procedure, we categorized the type of molecular interactions by examining whether Mo_A_ and Mo_B_ belonged to the ‘head’ (H) Moatoms or the ‘side’ (S) Mo-atoms. The ‘H’ Mo atoms were the two Mo atoms at each terminal of the ‘molecular’ MoS_2_, where the ‘S’ Mo atoms were the other non-terminal Mo atoms. These allowed us to categorize the types of molecular interactions as either head-to-head (HH), head-to-side (HS), or side-to-side (SS). For the analysis of perimeter-to-area (PtA) ratio, we first computed the ratio of the measured nanoribbon edge length against the measured nanoribbon area. For an insightful comparison, we compared the obtained ratio with that for an ideal nanoribbon, whose width and length followed that measured for the MoS_2_ nanoribbons. For example, for the ~4 nm wide nanoribbon obtained for the N ≥ 4 case shown in Figure 3a, the PtA ratio obtained experimentally was 2.3 ± 0.5 nm edge length per nm^2^ of the nanoribbon area, while the PtA ratio of an ideal, non-porous nanoribbon was 0.6 nm edge length per nm^2^ of the nanoribbon area. This results implied that the experimentally observed nanoribbons had ~3.8 times more edge length per nanoribbon area than an ideal, solid nanoribbon.

### Statistical Analysis

All statistical measurements were presented as mean ± SD, where SD is the standard deviation. The width distributions of the nanoribbon were subjected to Welch’s ANOVA test, well-suited for samples with unequal variances and sample sizes, implemented in Python’s pingouin package^89^.

## Supplementary Material

SI

## Data Availability

All data is available in the main text and supplementary information. Computed MoS structures on graphene and in the gas phase, the measured nanoribbon widths and angles, as well as the trajectories for MoS molecules reacting with one another are available at the Data Repository of the Max Planck Society (https://doi.org/10.17617/3.O1RHX5).
