# Supplementary material for: Controlled Formation of Nanoribbons and Their Heterostructures via Assembly of Mass-selected Inorganic Ions
Source: Adv Mater. Author manuscript; Available in PMC 2025 May 27. (PMC7617706; doi:10.1002/adma.202310817)
Supplement: SI [file EMS205803-supplement-SI.pdf]

Supplementary Information

Controlled Formation of Nanoribbons and Their Heterostructures via Assembly of Mass-selected Inorganic Ions

Xuejiao Zhang[*1], Vesna Srot[1], Xu Wu[1], Klaus Kern[1,2], Peter A. van Aken[1], Kelvin Anggara*[*1]*

Affiliations:
[1]*Max-Planck Institute for Solid-State Research, Stuttgart, DE-70569, Germany.*
[2]*Institut de Physique, École Polytechnique Fédérale de Lausanne, Lausanne, CH-1015, Switzerland.*

Corresponding author emails:
Xuejiao Zhang (Xj.Zhang@fkf.mpg.de)
Kelvin Anggara (k.anggara@fkf.mpg.de)

Ion	$[HS(MoS_3)_N]^{1-}$ $N \geq 4$	$[HS(MoS_3)_N]^{1-}$ $N \geq 5$	$[HS(MoS_3)_N]^{1-}$ $N \geq 6$	$[HS(WS_3)_N]^{1-}$ $N \geq 4$	Mixed $[HS(MoS_3)_N]^{1-}$ ($N \geq 6$) and $[HS(WS_3)_N]^{1-}$ ($N \geq 4$)
Current (pA)	~50	~30	~20	~20	~10

Table S1. Ion current observed for different deposited ions.

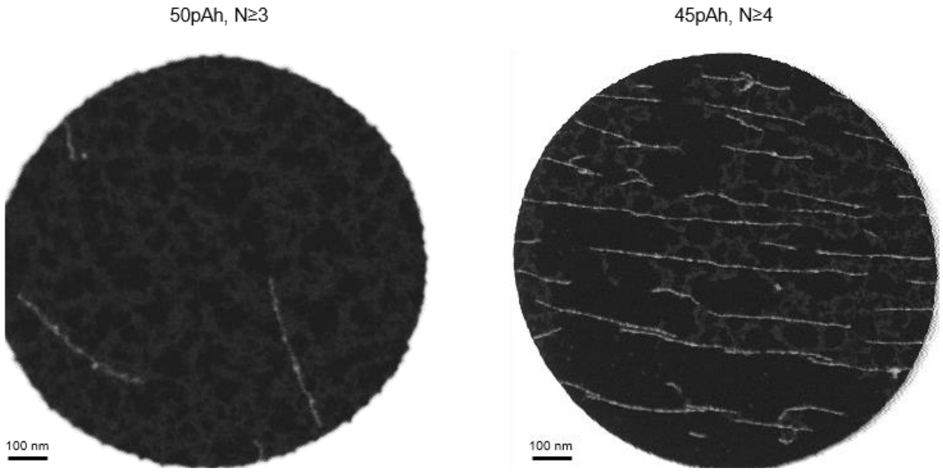

Figure S1. Graphene deposited with $[HS(MoS_3)_N]^{1-}$, where ($N \geq 3$), was observed with significantly fewer nanoribbons than ($N \geq 4$) samples with similar coverage. These results are understood to be due to the higher adsorption energies of heavier MoS ions allowing these ions to be retained on graphene long enough to form nanoribbons.

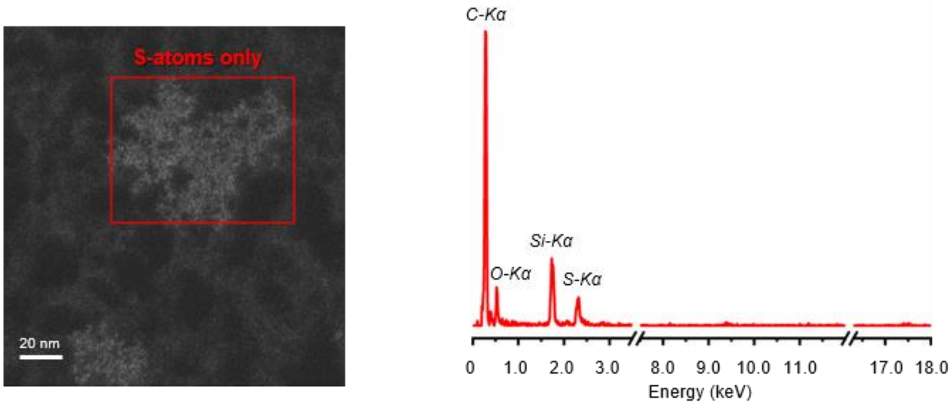

Figure S2. STEM-HAADF image and EDX spectrum of graphene deposited with $[HS(MoS_3)]^{1-}$ ions. Only S atoms were observed on graphene, as confirmed by EDX spectroscopy (Si signal comes from the SiN_x grid).

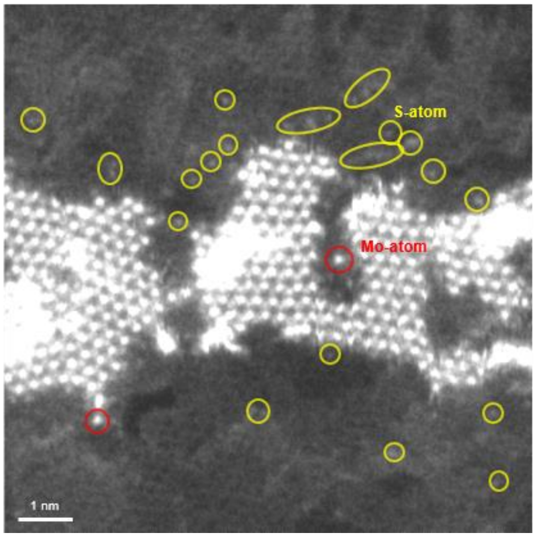

Figure S3. Individual Mo and S atoms observed around the MoS nanoribbon. The Mo adatoms are marked with red circles, while the S adatoms are marked with yellow circles. These adatoms can be explained by the electron transfer that occurs when the incident MoS ions approach the graphene surface to cause electron-induced dissociations, as we observed the formation of S adatoms when $HMoS_4^-$ was deposited on graphene (Figure S2). We expect that similar dissociation pathways exist for heavier MoS ions, such as $HMo_4S_{13}^{1-}$, $HMo_5S_{16}^{1-}$, $HMo_6S_{19}^{1-}$, and others.

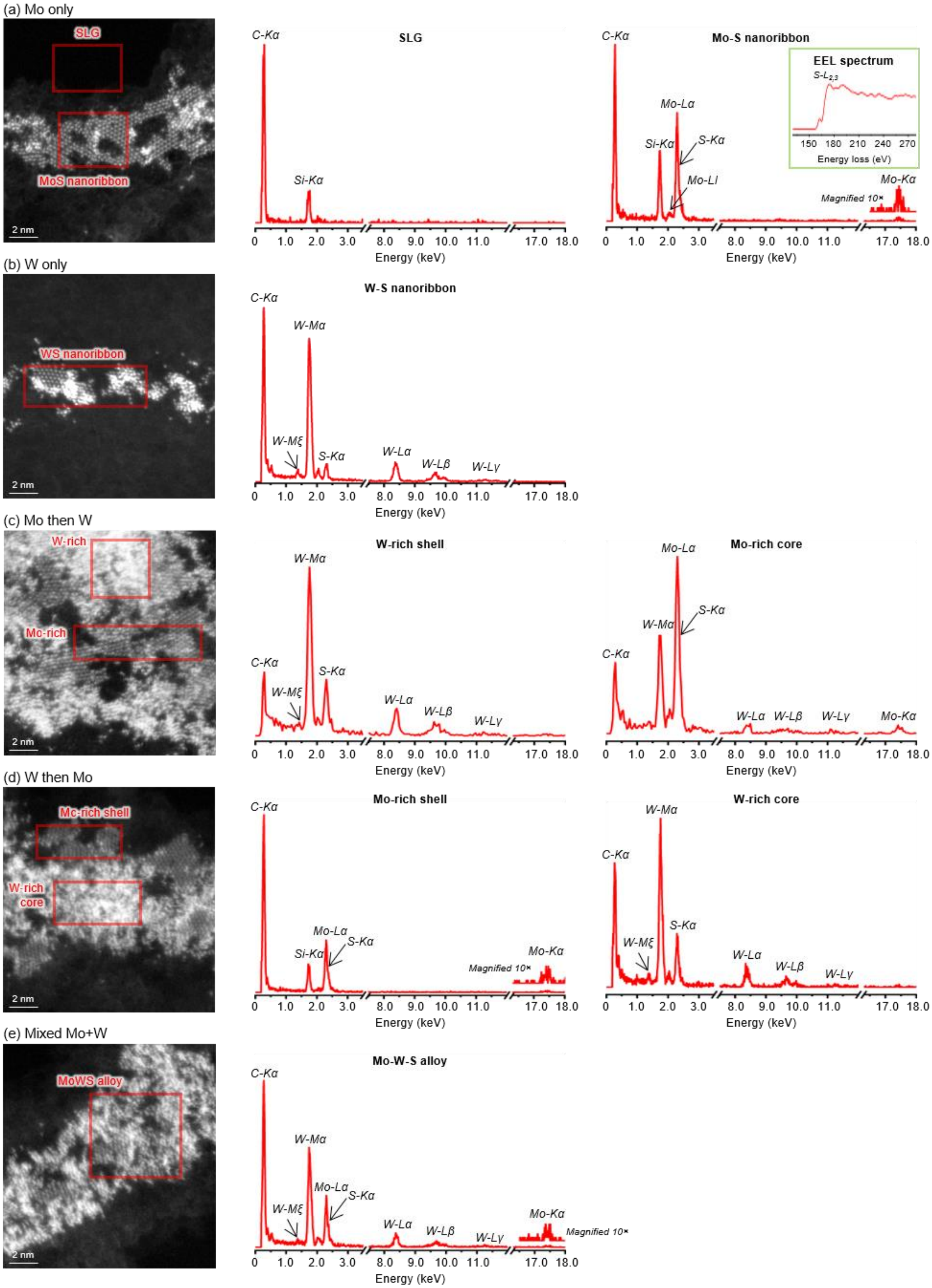

(a) Mo only
SLG
MoS nanoribbon
2 nm
SLG
C-Kα
Si-Kα
Energy (keV)
Mo-S nanoribbon
EEL spectrum
Energy loss (eV)
Mo-Lα
Si-Kα
S-Kα
Mo-Ll
Mo-Kα
Magnified 10×
(b) W only
WS nanoribbon
W-S nanoribbon
W-Mα
W-Mξ
W-Lα
W-Lβ
W-Lγ
(c) Mo then W
W-rich
Mo-rich
W-rich shell
Mo-rich core
(d) W then Mo
Mo-rich shell
W-rich core
(e) Mixed Mo+W
MoWS alloy
Mo-W-S alloy

Figure S4. EDX and EELS of the observed nanoribbons. EDX spectra performed on the areas marked in the given images confirm in (a) the presence of Mo and S atoms in the MoS_2 nanoribbons, in (b) W and S atoms in the WS_2 nanoribbons, in (c) W and S atoms in a W-rich shell, and Mo and S atoms in a Mo-rich core for the Mo-core W-shell nanoribbons, in (d) Mo and S atoms in a Mo-rich shell, and W and S atoms in a W rich core for the W-core Mo-shell nanoribbons; in (e) Mo, W, and S atoms in the MoWS alloyed nanoribbons. Due to the overlap of Mo and S peaks in EDX, we confirm the presence of S atoms in MoS by EELS, which shows the characteristic S-$L_{2,3}$ edges. The Mo-Kα peak is magnified by 10 times in several spectra due to the low signal count of the peak. The unit on the y-axis is a.u.

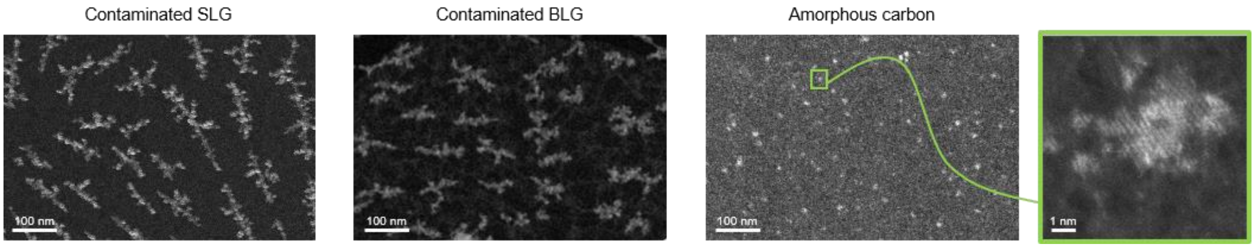

Figure S5. Deposition of MoS ions on different contaminated surfaces. Deposition of MoS ions on single-layer graphene (SLG) or bilayer graphene (BLG) contaminated with hydrocarbon impurities results in shorter nanoribbons with a higher degree of branching. Deposition of the MoS ions on amorphous carbon results in randomly shaped MoS islands.

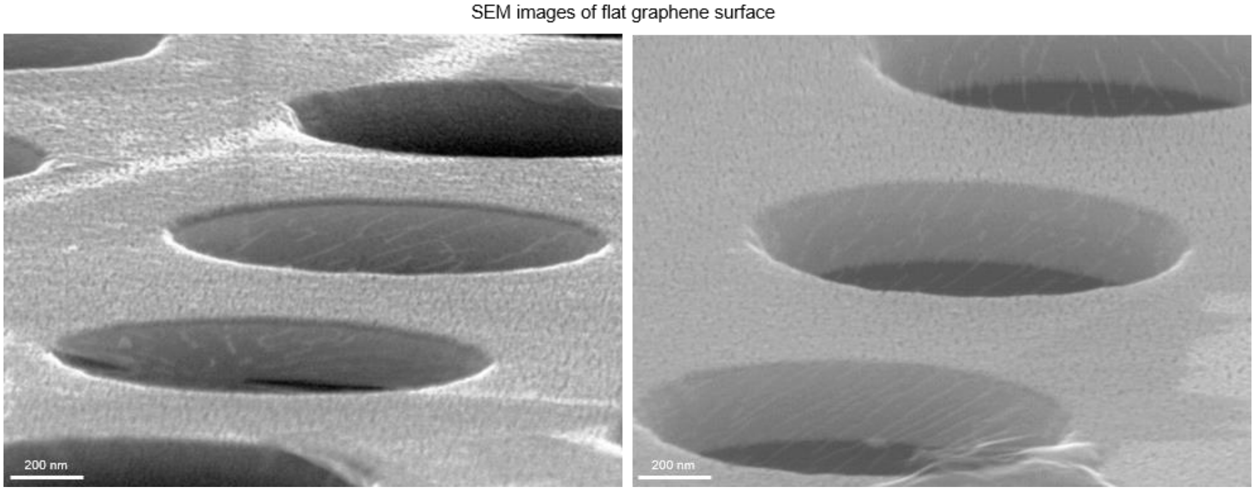

Figure S6. SEM images of MoS nanoribbons on single-layer graphene. SEM of the graphene sample imaged at high tilt angle (>70°) shows the absence of graphene ripples, confirming the flat graphene surface in our samples. The light grey lines on the graphene are the MoS_2 nanoribbons. Wavy graphene has been observed previously by SEM, as shown in Ref [54].

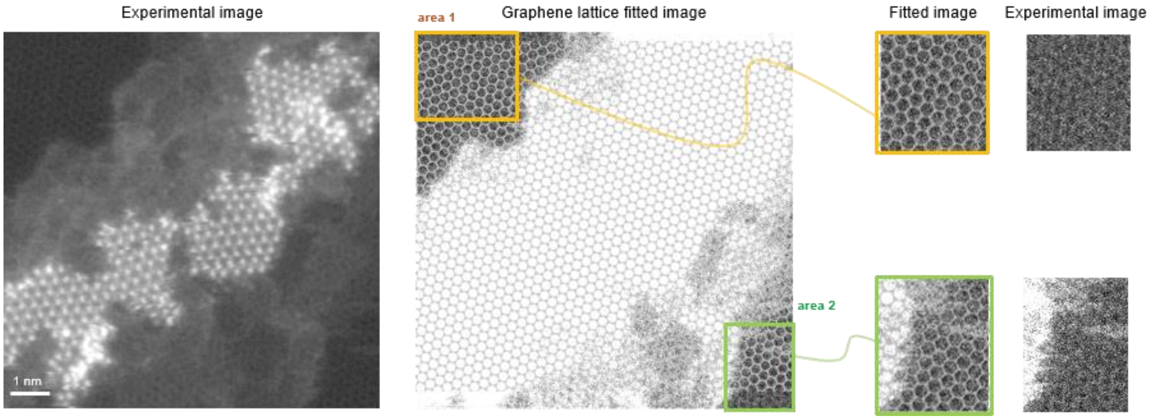

Figure S7. An example of STEM-HAADF image showing MoS_2 nanoribbons on lattice-resolved single-layer graphene. By analyzing the hexagonal lattice of the graphene on both sides of the nanoribbon, the presence of grain boundaries or line defects is ruled out.

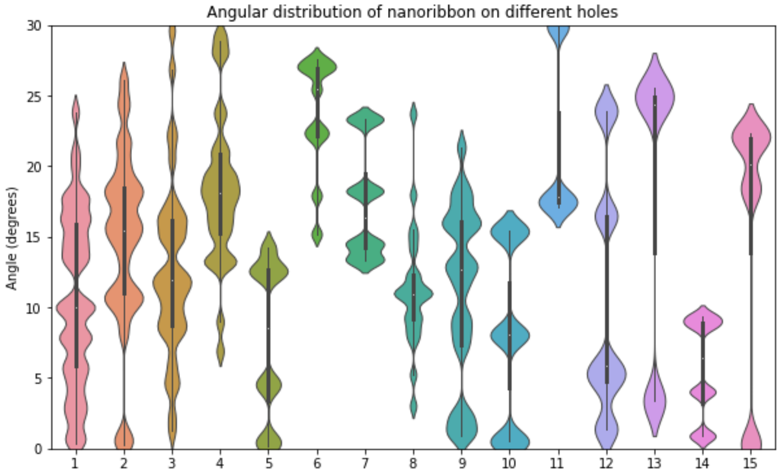

Figure S8. A violin plot obtained by Python's seaborn package (https://seaborn.pydata.org/) showing the nanoribbon orientation with respect to the six fold graphene lattice, showing that the nanoribbon orientation observed on different holes is highly variable. Due to the symmetry considerations, the data has been transformed to between 0 and 30 degrees (Data is available at https://doi.org/10.17617/3.O1RHX5).

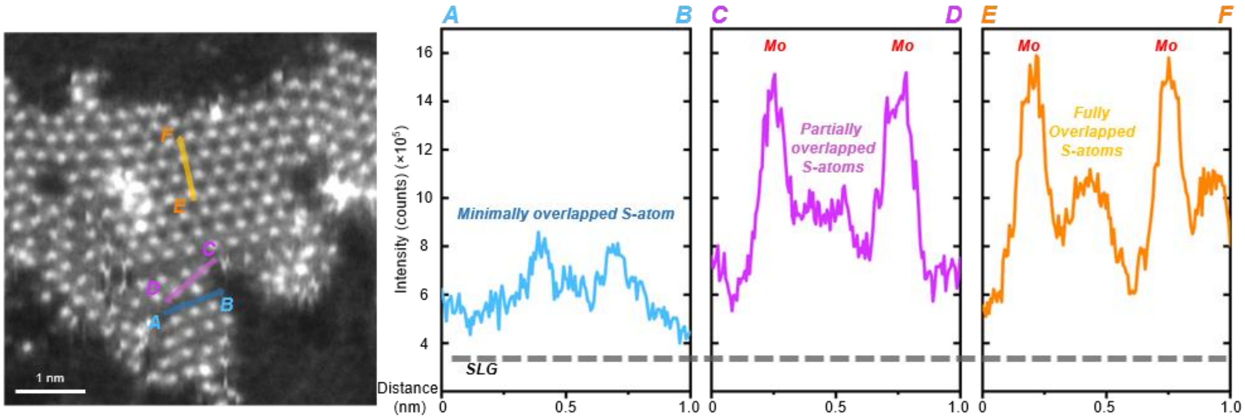

Figure S9. Comparison of three different S-atom contrasts in the nanoribbon. The three contrast intensity profiles are obtained by different S-atom geometries. A-B profile (obtained from the blue line in the left HAADF image) is the minimally overlapped S-atoms between two molecular species, the C-D profile (obtained from the purple line in the left HAADF image) is the partially overlapped S-atoms in the molecular species, and the E-F profile (obtained from the orange line in the left HAADF image) is the fully overlapped S-atoms in the condensed species. The dark grey dashed line in the profiles is the contrast intensity of single-layer graphene (SLG).

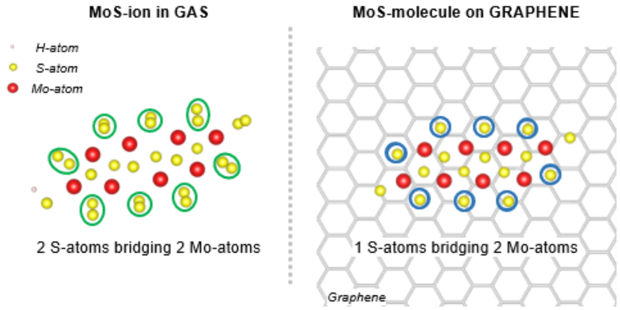

Figure S10. Comparison of the most stable structure of MoS ions in the gas phase and the structure of 'molecular' MoS_2 on graphene. Using Mo_8 as a model system, the most stable structure of $HMo_8S_{25}^{1-}$ in the gas phase is remarkably similar to the 'molecular' MoS_2 with the same number of Mo atoms. The main difference lies in the S atom bridging the edge Mo atoms: in the MoS ions, the two edge Mo atoms are bridged by two S atoms, whereas in the 'molecular' MoS_2, the two edge Mo atoms are bridged by one S atom.

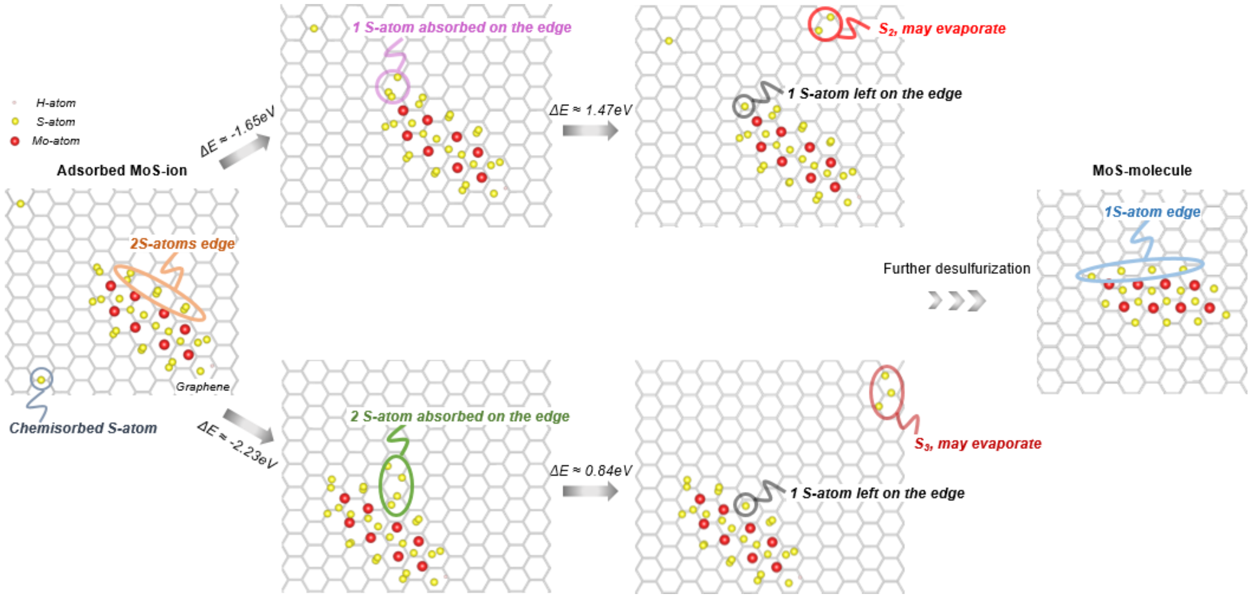

Figure S11. Calculated reaction pathways of adsorbed MoS ions transforming into 'molecular' MoS_2. Using a Mo_8 model system on graphene, our DFT calculations reveal two exothermic pathways by which the adsorbed MoS ions (HMo_8S_{25}) could transform into 'molecular' MoS_2 (Mo_8S_{16}). The pathways show a sequential attachment of chemisorbed S atoms to the adsorbed HMo_8S_{25}, followed by a detachment of S_2 or S_3 from the molecule, effectively converting the S_2 edge of the molecule into an S_1 edge. We assume that further $S_2 \rightarrow S_1$ conversion would convert the adsorbed MoS ions (HMo_8S_{25}) into 'molecular' MoS_2 (Mo_8S_{16}). Given the low adsorption energies of S_2 and S_3 physisorbed on graphene (E_{ads} = 0.30 and 0.42 eV, respectively) compared to the chemisorbed S atoms (E_{ads} = 1.84 eV), we expect the S_2 and S_3 molecules to desorb from the graphene at room temperature. The energy differences (ΔE) shown in the figure are the average potential energy differences from the chemisorbed S atom reacting with each S_2 site in the adsorbed MoS ions (HMo_8S_{25}) and from the dissociation of each S_3 or S_4 site in the molecule, respectively.

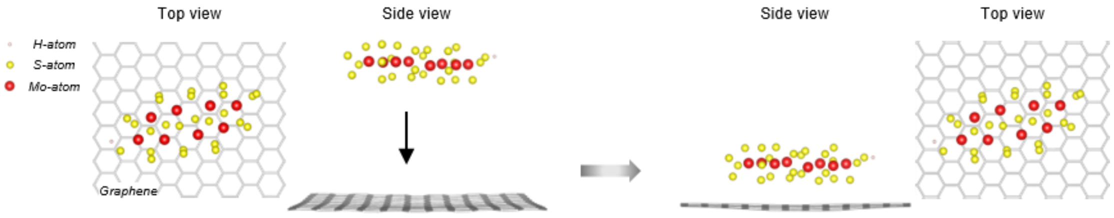

Figure S12. Calculated landing dynamics of MoS ions on single-layer graphene. MD calculations show that HMo_8S_{25} landing with 3 eV of kinetic energy on a single-layer graphene preserves the entire structure of the molecule on the surface. The MoS ions are calculated to physisorb on the graphene (E_{ads} = 1.95 eV) without changing the sp^2 hybridization of the C-atoms on graphene.

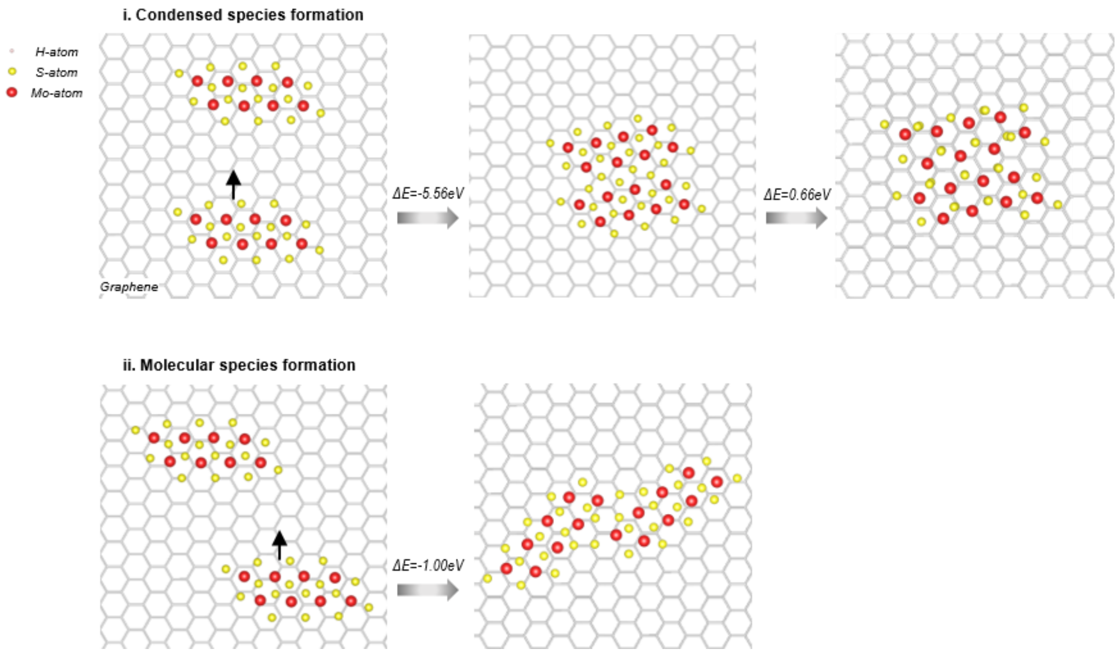

Figure S13. Calculated association dynamics of two 'molecular' MoS_2 (Mo_8S_{16}). MD calculations approximating thermal reactions of 'molecular' MoS_2 on graphene show two reactive outcomes: 'condensed' MoS_2 islands and coalesced 'molecular' MoS_2, in agreement with experimental observations. The cases shown in the figure illustrate the importance of the impact parameter (i.e., the miss distance between the projectile and the target) in determining the collision outcome. For an impact parameter close to zero (top panel), the molecules collide to form a 'condensed' 1T-MoS_2 island, which we expect to transform into 1H-MoS_2 islands on longer timescales. In contrast, at a larger impact parameter of ~9 Å (bottom panel), the molecules collide to form coalesced 'molecular' MoS_2.

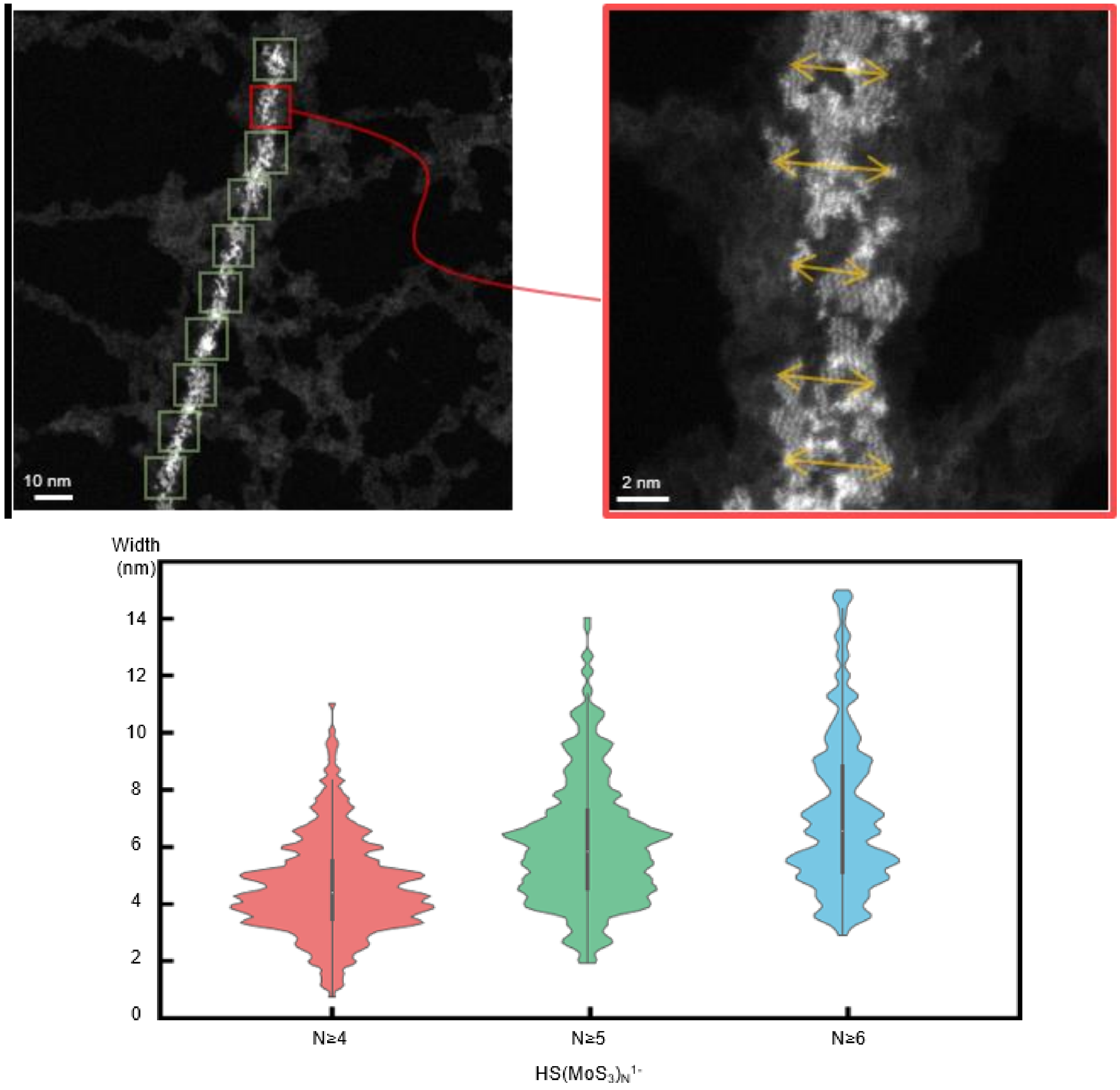

Figure S14. For every nanoribbon observed, the nanoribbon widths were manually measured every ~5 nm along the ribbon using high-magnification STEM-HAADF images. Violin plot of width distribution obtained by Python's seaborn package (https://seaborn.pydata.org/) showing the width distributions of nanoribbons obtained from depositing $[HS(MoS_3)_N]^{1-}$ ions with $N \geq 4$, $N \geq 5$ and $N \geq 6$ on graphene, respectively. The distributions were subjected to Welch's ANOVA test to yield p-values as low as 10^{-6}, indicating that the differences between these distributions are statistically significant. The sample sizes for $N \geq 4$, $N \geq 5$ and $N \geq 6$ are respectively 395 (from 7 nanoribbons imaged), 330 (from 11 nanoribbons imaged), and 220 (from 18 nanoribbons imaged) (Data is available at https://doi.org/10.17617/3.O1RHX5).

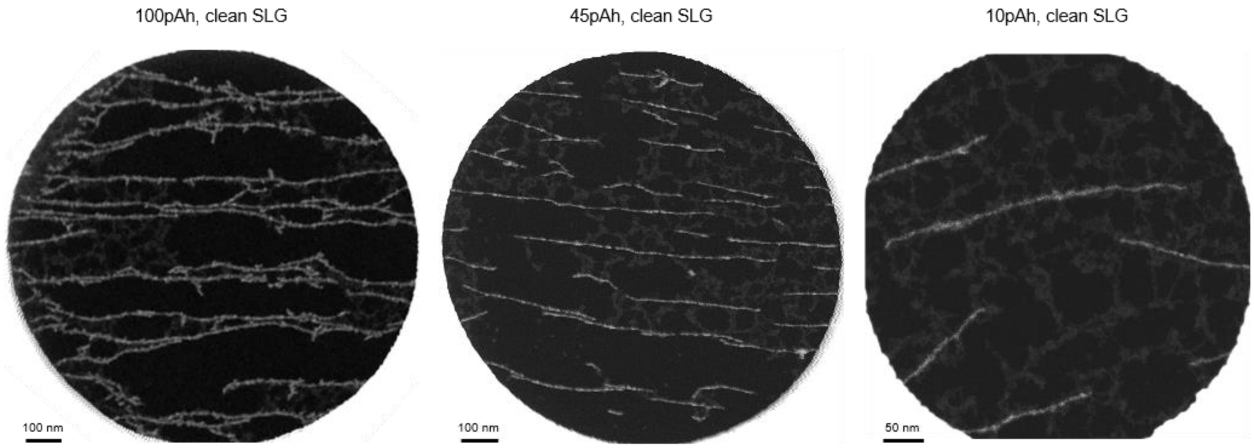

Figure S15. The length and numbers of nanoribbons on graphene depend on the amount of MoS ions deposited. Increasing the amount of MoS ions deposited on graphene leads to a higher number of nanoribbons and longer nanoribbons on the surface. The longest nanoribbons observed for 10, 45, and 100 pAh samples are ~300 nm, ~850 nm, and ~1200 nm respectively. In addition, the number of nanoribbons observed for 10, 45, and 100 pAh samples is ~31, ~45, and ~85 nanoribbons per μm^2 area examined, respectively. Here, we express the total deposited ions in picoampere hours (pAh), where 1 pAh (total charges for 1 pA current for one hour) corresponds to 3.6 nanocoulombs or ~2.3 × 10^{10} elementary charges.

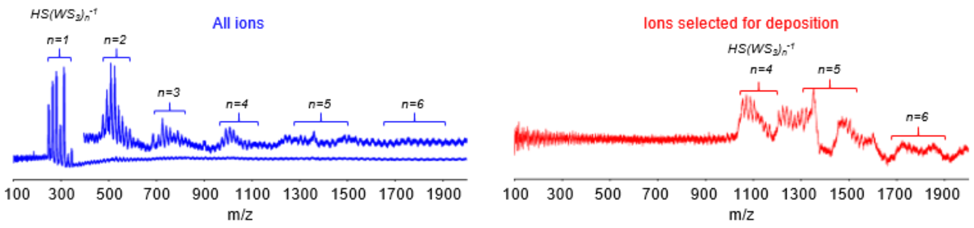

Figure S16. Time-of-flight mass spectrum of WS ions. $[HS(WS_3)_N]^{1-}$ ions obtained from the electrospray are characterized by their mass-over-charge (blue) before being mass-selected for surface deposition at N ≥ 4 (red). The multiple peaks observed are attributable to the different isotopes of the W atoms as well as the substitution of S atoms by O atoms.

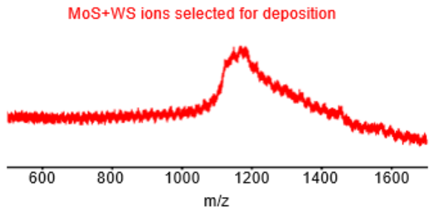

Figure S17. Time-of-flight mass spectrum of selected MoS and WS ions simultaneously deposited on graphene. The ions used for simultaneous Mo, W deposition include $[HS(MoS_3)_N]^{1-}$ ($N \geq 6$) and $[HS(WS_3)_N]^{1-}$ ($N \geq 4$).
